# Spatial Attention Reduces Burstiness in Macaque Visual Cortical Area MST

**DOI:** 10.1093/cercor/bhw326

**Published:** 2016-11-22

**Authors:** Cheng Xue, Daniel Kaping, Sonia Baloni Ray, B. Suresh Krishna, Stefan Treue

**Affiliations:** 1 Cognitive Neuroscience Laboratory, German Primate Center, Goettingen 37077, Germany; 2 Experimental Neurobiology, National Institute of Mental Health, Klecany 25067, Czech Republic; 3 Centre of Behavioural and Cognitive Sciences, University of Allahabad, Allahabad 211001, UP, India; 4 Faculty of Biology and Psychology, Goettingen University, Goettingen 37073, Germany; 5 Leibniz Science Campus Primate Cognition, Goettingen 37073, Germany

**Keywords:** attention, burstiness, monkey neurophysiology, visual cortex

## Abstract

Visual attention modulates the firing rate of neurons in many primate cortical areas. In V4, a cortical area in the ventral visual pathway, spatial attention has also been shown to reduce the tendency of neurons to fire closely separated spikes (burstiness). A recent model proposes that a single mechanism accounts for both the firing rate enhancement and the burstiness reduction in V4, but this has not been empirically tested. It is also unclear if the burstiness reduction by spatial attention is found in other visual areas and for other attentional types. We therefore recorded from single neurons in the medial superior temporal area (MST), a key motion-processing area along the dorsal visual pathway, of two rhesus monkeys while they performed a task engaging both spatial and feature-based attention. We show that in MST, spatial attention is associated with a clear reduction in burstiness that is independent of the concurrent enhancement of firing rate. In contrast, feature-based attention enhances firing rate but is not associated with a significant reduction in burstiness. These results establish burstiness reduction as a widespread effect of spatial attention. They also suggest that in contrast to the recently proposed model, the effects of spatial attention on burstiness and firing rate emerge from different mechanisms.

## Introduction

Attention is a critical component of sensory processing in organisms ranging from insects to humans (Carrasco 2011; Wiederman and O'Carroll 2013). It serves to preferentially allocate sparse processing resources to currently relevant sensory input, thereby privileging it over the remaining inputs. In humans and other primates, visual attention enhances the processing of task-relevant spatial locations and visual features (such as a particular motion direction or color) that leads to improved visual performance at these spatial locations and features (Desimone and Duncan 1995; Moore and Armstrong 2003; Bichot et al. 2015). The perceptual improvements induced by spatial and feature-based attention are accompanied by a range of neural effects that affect neuronal spike-rate (Desimone and Duncan 1995; Treue 2001; Bisley 2011), the temporal patterning of spike trains (Anderson et al. 2013), the mutual correlation between neurons (Cohen and Maunsell 2009, 2011a,2011b; Mitchell et al. 2009) and the local field potential (Fries 2009; Esghaei et al. 2015). These effects have been hypothesized to improve the sensory representation of attended stimuli by enhancing neural responses and reducing noise among neurons that represent the attended locations and/or features. Recently, it has been shown in V4, a key locus in the ventral stream of visual cortical information processing, that attention can also modulate aspects of neuronal firing patterns that operate on a fast timescale: burstiness, defined as the tendency of a neuron to discharge consecutive spikes at very short inter-spike intervals, decreases in the broad-spiking neurons of area V4 when spatial attention is directed into their receptive field (RF) (Anderson et al. 2013). Though the specific functional consequence of this attentional modulation remains unknown, the effect is intriguing, because the functional properties and neural utility of bursts in spike trains have been a topic of much speculation and interest (Bair et al. 1994; Krahe and Gabbiani 2004; Izhikevich 2007). A current and plausible hypothesis states that bursts enhance information transfer because neuronal inputs composed of closely spaced spikes are more efficient at driving postsynaptic neurons which act as coincidence detectors because of their short integration time constants (Lisman 1997). As pointed out by Anderson et al. (2013), this hypothesis predicts that to drive downstream neurons more efficiently, burstiness would increase when attention is directed towards a neuron's RF. However, the burstiness reduction observed indicates the opposite.

At present, it remains unclear if the effect of spatial attention on burstiness is restricted to the ventral pathway or even only V4 and whether it extends to other types of attention. Furthermore, though it has been recently proposed based on a computational model that the effects of spatial attention on burstiness and firing rate emerge from a common mechanism (Anderson et al. 2013), there is no empirical data on how the attentional modulation of burstiness relates to the well-known modulation of firing rate by attention. To address this, we performed and analyzed extracellular single-neuron recordings from the medial superior temporal area (MST) of two rhesus monkeys performing a spatial and feature-based attention task. Both shifting spatial attention into the RF and deploying feature-based attention to the preferred direction (relative to the non-preferred direction) enhanced the firing rate of MST neurons, as expected based on previous studies (Treue and Maunsell 1996; Treue and Martinez Trujillo 1999; Patzwahl and Treue 2009). In addition, spatial attention also led to a concurrent net reduction in burstiness, as reported earlier from V4. However, feature-based attention is not associated with a significant reduction in burstiness, though it did enhance firing rate. This absence of significant burstiness reduction cannot be explained by the smaller effect size of feature-based attention compared with spatial attention. Furthermore, the effects of spatial attention on firing rate and burstiness could be dissociated. Our results extend our understanding of the attentional effects on the temporal patterns of action potential discharge and support the idea that different types of attention may involve different physiological mechanisms.

## Materials and Methods

### Animal Use and Surgical Procedures

Data were collected from two male rhesus monkeys (*Macaca mulatta,* Monkey W, Monkey N, both 12-year-old males). Area MST was accessible through a recording chamber implanted over the parietal lobe based on a magnetic resonance imaging (MRI) scan (right hemisphere for Monkey W, left hemisphere for Monkey N). Each monkey was implanted with a titanium head holder to minimize head movements during the experiment. Both monkeys were seated in custom-made primate chairs and head-fixated during the experiment. All procedures were conducted in accordance with German laws governing animal care and approved by the district government of Oldenburg, Lower Saxony, Germany. Surgeries were conducted under general anesthesia and post-surgical care using standard techniques.

### Experimental Setup

The monkeys performed the tasks in a dimly lit room, with the only source of light being the display monitor. A custom computer program for experiment control, running on an Apple Macintosh PowerPC handled the stimulus presentation, eye-position control, as well as data collection and storage. Eye positions were monitored with a video-based eye tracker (ET49, sampling rate 60 Hz; Thomas Recording, Giessen, Germany). A CRT monitor placed at a distance of 57 cm from the monkey was used to display the visual stimulus at a refresh rate of 60 Hz and a spatial resolution of 40 pixels per degree. The monitor covered approximately 40° **×** 30° of visual angle.

### Electrophysiological Procedures

We recorded neuronal activity extracellularly using a three-channel microdrive system (Mini Matrix; Thomas Recording) and a Multichannel Acquisition Processor system (Plexon, Inc., Dallas, TX), running at a sampling rate of 40 kHz. Action potentials were sorted online (waveform window discrimination, Sort Client; Plexon Inc.) and recorded. MST was identified by referencing the recordings to the structural MRI and by the physiological properties of the recorded neurons (large RFs compared with MT and direction tuning to spiral motion; Graziano et al. (1994)). We recorded data from well-isolated neurons if their response to the preferred spiral motion direction was at least twice as high as the response to the null direction. Six recorded neurons were excluded from this population as we were unable to record at least three hit trials for each trial condition. Once a neuron was isolated, its RF was estimated by manually moving a static stimulus on the monitor while the monkey maintained his gaze on the fixation task. Once the RF was identified, a series of spiral motion stimuli were presented in the RF in sequence in order to determine the feature preference of the neuron. We used 12 spiral motion directions. The direction that elicited the highest response was taken as the “preferred direction” of the unit, while the opposite direction was taken as the “null direction.” After this phase of initial characterization, the monkeys performed different experimental tasks while the neuron's activity was recorded.

### Behavioral Task

**Figure 1.**
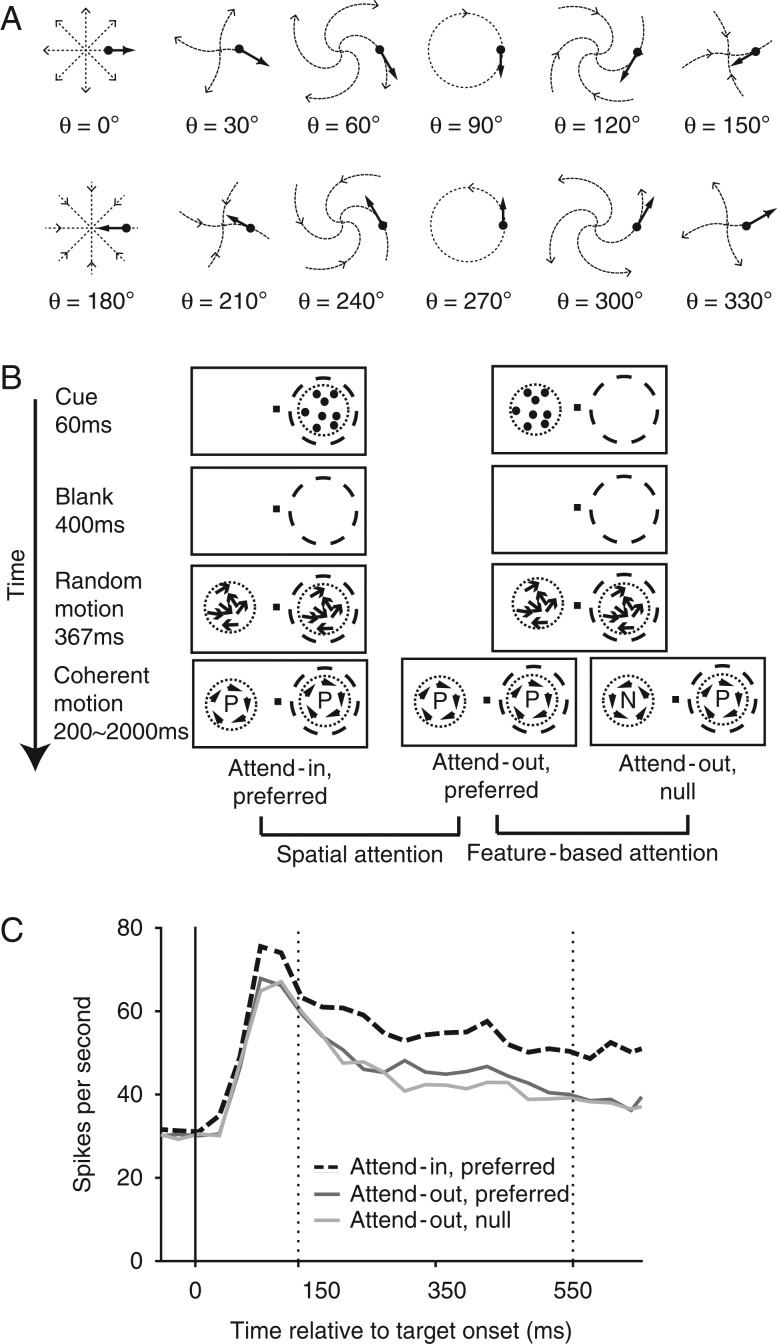
Behavioral task and neuronal responses. (*A*) Spiral motion stimuli. In each RDP stimulus, all dots move in directions that maintain a constant *angle θ* with the radial axis (see the thick black arrows), which defines the “spiral direction” of the RDP. The direction that elicited the highest response in a given neuron was taken as the “preferred direction” of the unit, while the opposite direction was taken as the “null direction.” (*B*) Trial sequence. Once the monkey depressed a lever and foveated the central fixation point (black square), a spatial cue (stationary RDP) briefly appeared either in or outside the RF of the recorded neuron (dashed circle). After a blank interval, two RDPs in non-coherent motion were presented. After 367 ms both stimuli became fully coherent, preferred (clockwise rotation in this example) or null (anti-clockwise rotation in this example) direction motion patterns. The monkey had to respond within 400 ms to a speed change in the cued stimulus (the “target”) to correctly complete the trial. The horizontal brackets indicate the conditions that were compared to establish the modulation by spatial or feature-based attention, respectively. (*C*) Average PSTH across our population of 100 MST neurons, with time relative to target and distractor onset (solid vertical line) in all three attentional conditions. Vertical dotted lines indicate the start and end of the analysis period.

We analyzed three different conditions from the cued detection task in this study. In cued detection trials, the monkeys had to respond to a speed change in 1 of 2 spiral motion stimuli (the target, identified by a preceding stationary cue presented at the same location) while ignoring similar changes in the other stimulus (the distractor). The spiral motion stimuli were random dot patterns (RDPs) in which the motion direction of all dots in a given RDP maintains a constant angle with the radial axis (Fig. 1*A*). MST neurons are known to be tuned for this “spiral direction” (Graziano et al. 1994). The RDPs had a diameter of 4° of visual angle and a dot density of 8 per square degree. The luminance of the dots was 75 cd/m^2^ on a gray background of 35 cd/m^2^.

The monkey started each trial by touching a lever and directing its gaze onto a central fixation point (0.2° × 0.2°). Throughout the trial, the monkeys were required to maintain their gaze within 1.8 degrees of the fixation point, or the trial was aborted. After 150 ms from the start of the trial, a static RDP was presented as a spatial cue for 67ms. After cue offset, a 400 ms blank period followed. The blank period ended with the onset of two zero-coherence spiral motion RDPs: one at the cued target location and the other at a location symmetrically opposite to it (i.e. reflected around the fixation point). After 367 ms, both RDPs turned into fully coherent spiral motions; this time point was defined as the target onset. The monkey had to respond within 400 ms (by releasing the lever) to a change in speed of the target RDP. At the same time, the monkey had to ignore any changes in the distractor, that is, the RDP at the uncued location. Each correctly completed trial was rewarded with juice. The target speed change time was randomly chosen for each trial from between 250 and 2500 ms after target onset.

For this study, we analyzed three behavioral conditions adapted from Treue & Martinez-Trujillo (1999) to determine the effects of spatial and feature-based attention: the *attend-in preferred* condition, the *attend-out preferred* condition, and the *attend-out null* condition. In all three conditions, the RDP in the RF moved in the preferred direction of the neuron. In the attend-in preferred condition, the RDP inside the RF was the target and the distractor RDP (outside the RF) also moved in the preferred direction. In the attend-out preferred and the attend-out null conditions, the RDP outside the RF was the target and moved in the preferred direction for the attend-out preferred condition and in the null direction for the attend-out null condition. Comparing neuronal responses in the attend-in preferred and attend-out preferred conditions isolates the effects of spatial attention, while comparing attend-out preferred to attend-out null isolates the effects of feature-based attention. Trials from the three conditions were performed in an interleaved manner.

### Data Analysis

All data analysis was performed using custom software in MATLAB R2015a (MATLAB Inc., Natick, MA). We included data from all neurons that showed a tuning for spiral motion direction, with the preferred direction position-invariant, that is, unaffected by placing the spiral motion at different positions within the RF (Graziano et al. 1994). We only included correctly performed trials in our analysis. Peri-stimulus time histograms (PSTHs) in Figure 1*C* were calculated using non-overlapping 30 ms bins. The mean activity for each neuron across trials was first calculated and then these mean PSTHs for individual neurons were averaged across neurons to obtain the displayed PSTHs.

### Burst Analysis

Burstiness was estimated for each neuron and each task condition, during an analysis period from 150 to 550 ms after target (and distractor) onset. We picked 150 ms as the start of the analysis window to exclude the transient activity induced by the coherent motion onset, and 550 ms as the end of the analysis window to ensure enough trials for the burstiness calculation where no motion change occurred in either the target or distractor RDP within the analysis window. We selected for analysis all correctly completed trials with neither a distractor speed change nor a target speed change during the analysis period. Only neurons with at least three such trials for each attentional condition were included. To quantify burstiness, we used the same approach described in 2 earlier studies (Compte et al. 2003; Anderson et al. 2011). For a set of trials from each neuron and each attentional condition, we first calculated the mean autocorrelation function (ACF). We then calculated a shuffle predictor defined as the cross-correlation function across all pairs of trials in the set. To obtain a normalized ACF, we subtracted the mean of the shuffle predictor from the mean ACF and normalized the difference with the standard deviation of the shuffle predictor. Burstiness was then defined as the average height of the normalized ACF for time lags from 1 to 4 ms. This burstiness measure is also partially similar to that in Anderson et al. (2013), with the difference that Anderson et al. (2013) normalized by the mean shuffle predictor (rather than its standard deviation) and further multiplied the normalized ACF by the impulse response of a band-pass filter (10–40 Hz) and integrated the result to obtain a burstiness value. The Anderson et al. (2013) procedure calculates burstiness by computing a weighted sum of the normalized ACF between 1 and 11 ms, 33 and 46 ms, 55 and 77 ms, and so on until 256 ms, and subtracts a weighted sum of the ACF between 11 and 33 ms, 46 and 55 ms, 77 and 92 ms, and so on until 256 ms. Since this decaying and roughly sinusoidal weighting function has a band-pass frequency spectrum from 10 to 40 Hz, this also has the effect of integrating the Fourier transform of the ACF between 10 and 40 Hz. Though this procedure appears quite different from the Anderson et al. (2011) procedure that we use, the burstiness values it generates are highly correlated with the ones generated by the Anderson et al. (2011) procedure, and our interpretations and conclusions remain the same using either measure (see Results).

### Quantifying Attentional Modulation

We quantified the magnitude of attentional modulation of firing rate using a very common attentional index, defined as the difference of values between attentional conditions normalized by their sum. Specifically, the attentional index of spatial attention on firing rate (denoted by FR) was calculated as:

$${\mathrm{AI}}_{\mathrm{FR}}^{\mathrm{spatial}}={\mathrm{FR}}_{\mathrm{in}}-{\mathrm{FR}}_{\mathrm{out}}/{\mathrm{FR}}_{\mathrm{in}}+{\mathrm{FR}}_{\mathrm{out}}$$

where in and out refer to the conditions with spatial attention into and outside the RF (with both RDPs always moving in the preferred direction).

Similarly, the attentional index of feature-based attention on firing rate was calculated as:

$${\mathrm{AI}}_{\mathrm{FR}}^{\mathrm{feature}}={\mathrm{FR}}_{\mathrm{pref}}-{\mathrm{FR}}_{\mathrm{null}}/{\mathrm{FR}}_{\mathrm{pref}}+{\mathrm{FR}}_{n\mathrm{ull}}$$

where pref and null refer to attention to the preferred or null direction RDP outside the RF (with the preferred direction distractor RDP inside the RF).

Unlike firing rate, burstiness values using our measure can have values below 0, and the attentional index as defined above only works for non-negative values. We therefore simply use the difference of burstiness values between attentional conditions to quantify the attentional effect on burstiness. Finally, for both firing rate and burstiness, we report the averages using medians (after converting the median attentional index back to a percentage value) and use the Wilcoxon signed-rank test to assess statistical significance. We use the Kendall rank correlation to measure potential associations and determine statistical significance.

### Trial-Swap Analysis

To determine whether changes in firing rate could be dissociated from changes in burstiness, we also performed an analysis within individual neurons where for each neuron, we created new data sets by exchanging trials with similar firing rate between the attend-in preferred and attend-out preferred conditions. The goal of this trial-swap was to exchange as many trials as possible (with similar firing rates) between the attend-in and attend-out conditions, so that the mean firing rates of the 2 conditions only changed minimally, and even this minimal change always led to a greater enhancement of firing rate by spatial attention. Specifically, we first sorted the trials in the attend-in and attend-out conditions by their spike count. To choose trials for swapping, we created 2 subsets of trials: for Subset 1, we picked the N least spiking trials from the condition with higher mean firing rate, and for Subset 2, we picked the N most spiking trials from the condition with lower firing rate. N was chosen to be the largest number that ensured that the subset from the attend-in condition has lower mean firing rate than the subset from the attend-out condition. Chosen in this manner, swapping Subset 1 with Subset 2 retained or enhanced the attentional index for each neuron (Fig. 3*C*) and therefore predicted a larger reduction for burstiness as well, if the attentional effect on burstiness was coupled to that on firing rate.

### Waveform Duration Calculation

We recorded each spike waveform over a 800ms window with a sampling rate of 40 kHz. For each cell, we first normalized the height of the spike waveforms by calculating its z-score relative to the average of the waveform over time. The normalized waveforms were then aligned to the trough of each waveform, averaged to obtain a mean waveform and then interpolated to a time resolution of 1μs (using the MATLAB “interp1” function, “spline” mode). Waveform duration was then defined as the time duration between the trough and the following peak of the interpolated mean waveform.

## Results

We investigated the burstiness of 100 MST neurons from 2 monkeys (44 neurons for Monkey N, 56 neurons for Monkey W) in 3 attentional conditions (Fig. 1*B*), in which the monkeys were cued to attend to the spatial location and motion direction of a target stimulus in the presence of a second, distractor stimulus. In all 3 conditions, the physical stimulus within the neuron's RF was a RDP moving in the preferred direction. Monkey N correctly responded to the target change 92.8% of the time (hit rate), and missed the remaining 7.2 % of changes; releases before the target change occurred on 6.8% of trials (early release rate). Monkey W had a hit rate of 93.4% and an early release rate of 6.5%. The mean reaction times were 333 ms (standard deviation: 41 ms) for Monkey N and 319 ms (SD = 56ms) for Monkey W.

### Both Spatial and feature-based attention Modulate Firing Rate

**Figure 2.**
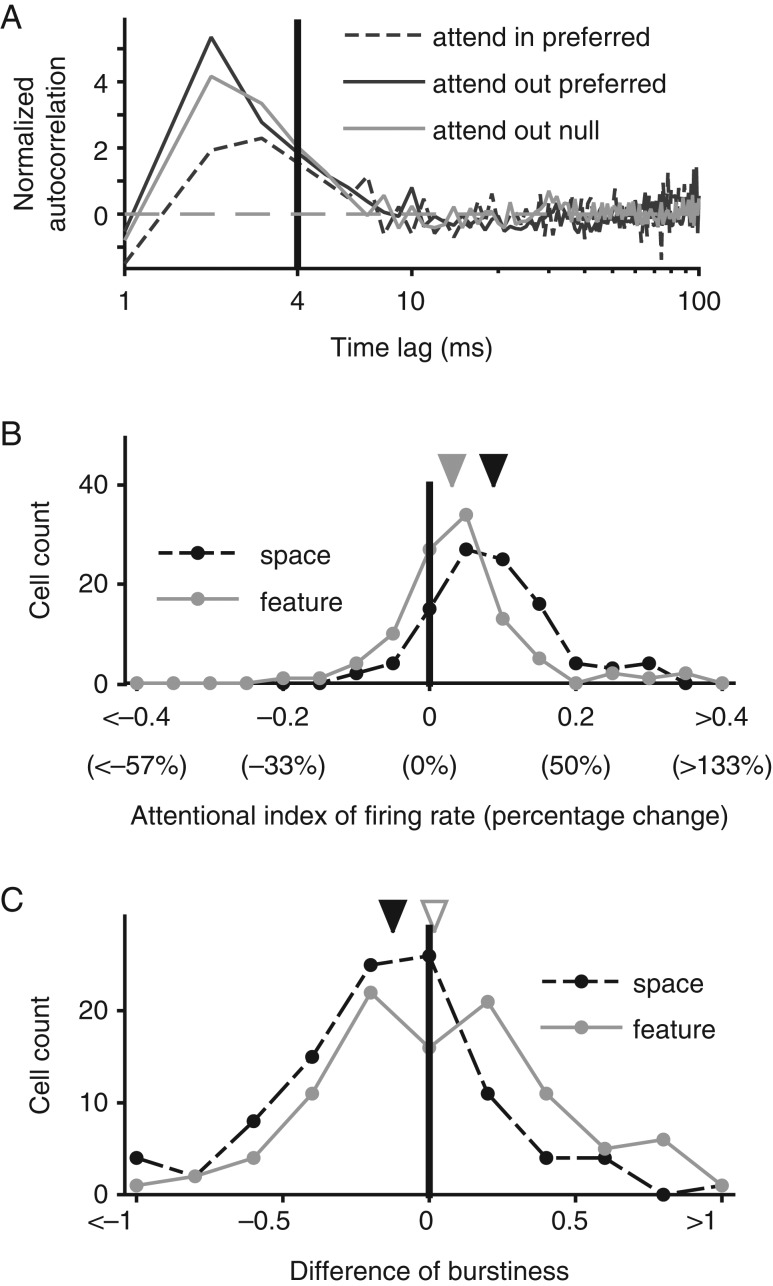
Spatial and feature-based attentional modulation of firing rate and burstiness. (*A*) The normalized autocorrelation of an example MST neuron in different attentional conditions. The *y*-axis and the vertical solid line at 4 ms time lag demarcate the range of time lags used to estimate the burstiness index. Burstiness is defined as the average height of the normalized autocorrelation for the time lags between 1 and 4 ms. The horizontal dashed line indicates the normalized autocorrelation for a Poisson spike train. (*B*) Distribution of attentional indices of firing rates with spatial attention (black dashed line) and feature-based attention (gray solid line). (*C*) Distribution of attentional indices of burstiness for spatial attention (black dashed line) and feature-based attention (gray solid line). In *B* and *C*, black and gray triangles indicate median index values for spatial and feature-based attention, respectively. Filled triangles indicate significant deviations from zero (signed-rank test, see Materials and Methods), open triangles indicate lack of significance. *X*-axis values in brackets represent percentage changes: positive values are increases and negative values are reductions.

When both RDPs moved in the preferred direction of the recorded neuron, comparing the responses when the RDP in the RF was the target to that when it was the distractor enabled us to examine the effects of spatial attention. Similarly, when the distractor was in the RF (and moved in the preferred direction), comparing the responses when the target RDP outside the RF moved in the preferred direction to that when it moved in the anti-preferred direction enabled us to examine the effects of feature-based attention. The average population PSTH (Fig. 1*C*) shows a clear enhancement of the firing rate by both spatial attention (slashed black curve compared with solid black curve) and feature-based attention (solid black curve compared with solid gray curve). Similarly, the attentional index of firing rate (Fig. 2*B*) shows a clear enhancement by both spatial and feature-based attention: the median increase in firing rate for spatial attention is 19.4% (*P *< 0.0001) in the overall population (Monkey N: 16.0%, *P *< 0.0001, monkey W: 21.8%, *P *< 0.0001) and the median increase in firing rate for feature-based attention is 6.7% (*P *< 0.0001) in the overall population (Monkey N: 6.6%, *P *< 0.0001, Monkey W: 6.7%, *P *= 0.005). The effect of spatial attention on firing rate is significantly greater than that for feature-based attention (overall population: *P* = 0.0002, 0.0001, Monkey N: *P *= 0.04, Monkey W: *P =* 0.002).

### Spatial Attention Significantly Reduces Burstiness; feature-based attention Does not

Spatial attention clearly reduces burstiness in our population of MST neurons. As depicted for an example neuron (Fig. 2*A*), the normalized ACF shows a clear reduction in height when spatial attention was directed into the RF, compared with when it was directed outside the RF (black dashed curve compared with grey curve). In the population, spatial attention led to a median reduction in burstiness of 0.105 in the overall population (*P *= 0.0001; Monkey N: median = −0.091, *P *= 0.006; Monkey W: median = −0.114, *P *= 0.005). However, feature-based attention did not significantly reduce burstiness in either of the 2 monkeys. It led to an increase in burstiness in Monkey W (median = 0.095, *P *= 0.04), and no significant effect on burstiness in Monkey N (median = −0.117, *P *= 0.1); this difference between the monkeys was statistically significant (rank sum test, *P *= 0.02). This was true even if the analysis window's beginning was shifted to 300 ms following RDP onset, in order to account for the delayed emergence of feature-based attention (black vs. gray curves in Fig. 1*C*): Again, Monkey W showed a significant increase (median = 0.082, *P *= 0.04) and Monkey N showed no significant effect (median = −0.055, *P *= 0.2). A direct pairwise comparison showed that spatial attention led to a larger reduction in burstiness than feature-based attention in both monkeys, but the effect was not significant in Monkey N (median additional effect with spatial attention in Monkey W = −0.136, *P *= 0.008; in Monkey N = −0.062, *P *= 0.6).

These conclusions do not depend on our specific implementation of the burstiness measure: using the burstiness measure from Anderson, Mitchell and Reynolds (2013), spatial attention again reduced burstiness (overall population: median reduction = 0.042, *P *< 0.0001; Monkey N: median reduction = 0.025, *P *= 0.007; Monkey W: median reduction = 0.066, *P *= 0.0006), and there was no significant effect on burstiness with feature-based attention (overall population: median increase = 0.002, *P *= 0.7; Monkey N: median increase = 0.001, *P *= 0.8; Monkey W: median increase = 0.023, *P *= 0.5).

### Burstiness and Firing Rate Modulation by Spatial Attention Are Uncorrelated

**Figure 3.**
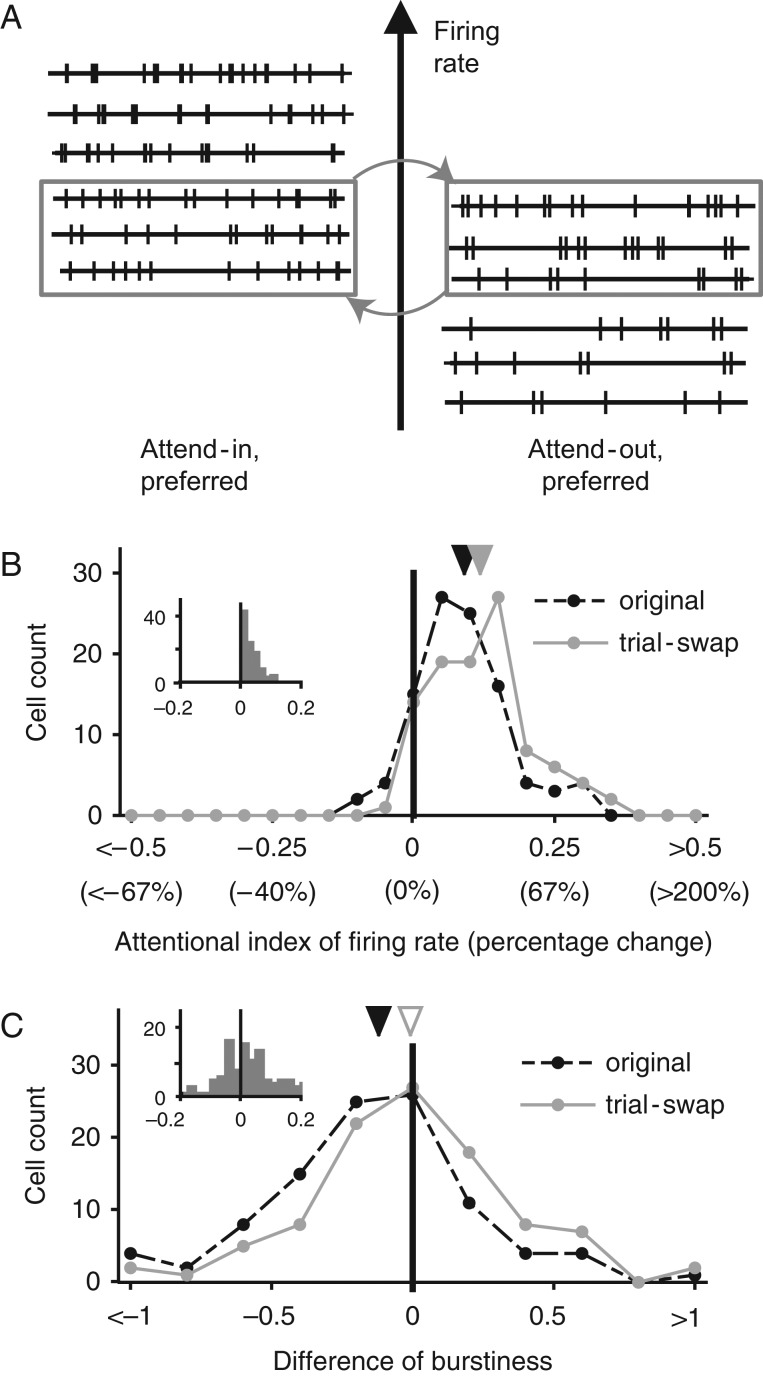
The reduction in burstiness by spatial attention can be dissociated from the firing rate increase. (*A*) Illustration of the trial-swap process. For each neuron, trial subsets with similar mean firing rates were swapped between spatial attention conditions. See Materials and Methods for details. (*B*) Distribution of attentional indices of firing rate across the MST population with spatial attention before (black dashed line) and after (gray solid line) swapping trials with similar firing rate. The inset shows the distribution of pairwise differences between attentional indices before and after the trial-swap. Trial-swaps were done conservatively, such that any change in the attentional index of firing rate was an increase. (*C*) Distribution of attentional indices of burstiness with spatial attention before (black dashed line) and after (gray solid line) swapping trials. Black and gray triangles indicate median index values for spatial and feature-based attention, respectively. Filled triangles indicate significant deviations from zero (signed-rank test, see Materials and Methods), open triangles indicate lack of significance. The inset shows the distribution of pairwise differences between attentional indices after and before trial-swap. The burstiness reduction effect disappeared after the trial-swap.

A recent computational model proposed that firing rate increases and burstiness reductions by spatial attention emerge via a common mechanism. A firing rate increase may also lead to a reduction in our burstiness measure in the presence of a refractory period (see Discussion). However, we find that the burstiness reduction by spatial attention can be dissociated from the concurrent firing rate increase. The effects of spatial attention on firing rate and burstiness were not correlated in the population of recorded neurons: the correlation coefficient between the change of firing rate and change of burstiness with spatial attention was −0.081 (*P *= 0.2) in the overall population, −0.042 (*P *= 0.7) in Monkey N, and −0.119 (*P *= 0.2) in Monkey W. We also performed an analysis within individual neurons, where for each neuron, we created new data sets by exchanging trials with similar firing rate between the attend-in and attend-out conditions (see Materials and Methods for detailed algorithm). The goal of this trial-swap was to exchange as many trials as possible (with similar firing rates) between the attend-in and attend-out conditions, so that the mean firing rates of the two conditions only changed minimally, and even this minimal change always led to a greater enhancement of firing rate by spatial attention (Fig. 3*C*). We reasoned that if the burstiness effect of attention was linked to the firing rate, then exchanging trials with similar firing rate between the two attentional conditions in this manner would either slightly enhance the attentional reduction of burstiness or leave it unchanged. However, if the burstiness reduction in the attend-in condition was linked to the attentional state (and not the firing rate), then exchanging trials between the attend-in and attend-out conditions would substantially reduce the burstiness reduction by attention. Our results support this latter hypothesis. As expected from the choice of trials, trial-swap slightly increased the median attentional index of firing rate: the median increase in the attentional index of firing rate after the swap was 0.023 in the overall population, 0.017 in Monkey N, and 0.034 in Monkey W, and every neuron showed a slight increase. However, the reduction of burstiness by spatial attention was no longer statistically significant after the trial-swap: the median difference in burstiness between the attend-in preferred and attend-out preferred conditions after the trial-swap was −0.008 (*P *= 0.8) in the overall population, −0.008 (*P *= 0.3) in Monkey N, and 0.043 (*P *= 0.5) in Monkey W (the values before the trials swap were −0.105 in the overall population, −0.091 in Monkey N, and −0.114 in Monkey W, as reported above). A pairwise comparison within neurons also indicated that there was a reduction in the effect of spatial attention on burstiness as a result of the trial-swap, though this reduction was only statistically significant in one monkey (median reduction in the burstiness differences between attend-in preferred and attend-out preferred conditions due to the trial-swap was 0.091, *P *= 0.03 in the overall population, 0.045, *P *= 0.6 in Monkey N, and 0.132, *P *= 0.02 in Monkey W). Overall, these results indicate that the burstiness reduction by spatial attention can be dissociated from its effects on firing rate.

### Differences in Burstiness Modulation by Spatial and Feature-based Attention Are not Confounded by Attentional Effect Size

**Figure 4.**
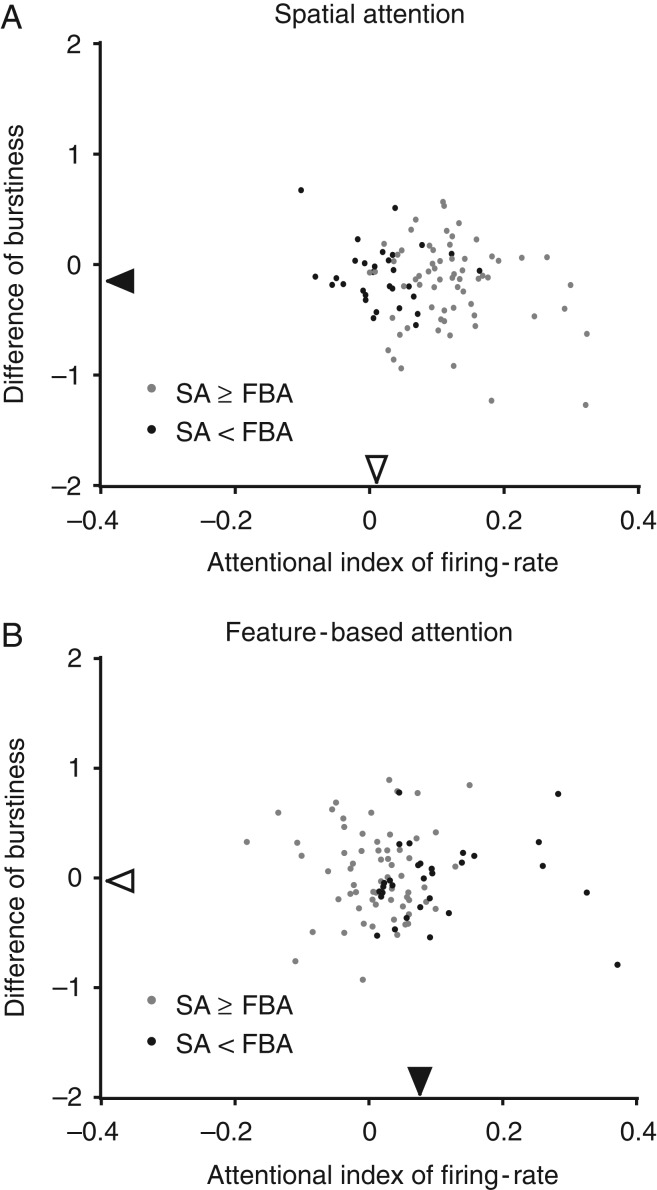
The lack of significant burstiness reduction by feature-based attention cannot be explained by the smaller effect size of FBA. (*A*) The effects of spatial attention on firing rate (abscissa) and burstiness (ordinate). Black dots represent neurons with a larger firing rate effect of feature-based attention (‘FBA’ in the figure) than of spatial attention (‘SA’ in the figure), while gray dots represent the remaining neurons. Both subpopulations (black and gray dots) showed a significant burstiness reduction but no correlation between firing rate and burstiness modulation (see text). In the figure, SA refers to spatial attention and FBA refers to feature based attention. (*B*) The effects of feature-based attention on firing rate and burstiness (plotting conventions as in *A*). In both panels, the black triangles indicate median index values for black dots on the respective axis; Filled triangles indicate significant deviations from zero (signed-rank test, see Materials and Methods), open triangles indicate lack of significance. Neither subpopulation (black and gray dots) showed a significant burstiness reduction or a correlation between firing rate and burstiness modulation (see text).

Both spatial and feature-based attention are associated with increases in the firing rate of MST neurons, in line with previous reports. We showed above that spatial attention was associated with a significant reduction of burstiness, while feature-based attention did not significantly reduce burstiness: feature-based attention actually increased burstiness significantly in Monkey W and led to a non-significant effect in Monkey N. However, one could argue that the lack of a significant burstiness reduction with feature-based attention may simply be a result of the smaller effect of feature-based attention (evidenced by a 6.7% median increase in firing rate) compared with spatial attention (with a 19.4% increase). We therefore examined the relationship between the attentional effects on firing rate and burstiness in the spatial and feature-based attention conditions (Fig. 4). There was no significant correlation between the attentional effects on firing rate and burstiness in either condition (spatial attention: *τ* = −0.031 (*P *= 0.6) in the overall population, *τ* = −0.011 (*P *= 0.9) in Monkey N, and *τ* = −0.068 (*P *= 0.5) in Monkey W; feature-based attention: *τ* = 0.021 (*P *= 0.8) in the overall population, *τ* = −0.011 (*P *= 0.9) in Monkey N, and *τ* = −0.068 (*P *= 0.5) in Monkey W), indicating that the reduction of burstiness by spatial attention could not be explained by its larger effect on firing rate. Similarly, no significant correlation was found when using the difference of firing rates as the measure instead of the attentional index (all *P*-values **≥**0.5). Furthermore, we examined the effect of spatial attention on burstiness in neurons where the spatial attentional indices of firing rate were smaller than the median feature-based attention index of firing rate (which equals 0.0323, and is also denoted by the filled gray triangle in Fig. 2*A*; the median index is 0.0321 in Monkey N, 0.0323 in Monkey W). Even within this subset of cells, spatial attention was associated with a reduction in burstiness (two monkeys pooled: median = −0.104, *P* = 0.02, *n* = 24; Monkey N: median = −0.154, *P* = 0.06, *n* = 10; Monkey W: median = −0.087, *P* = 0.03, *n* = 14). Consistent with the lack of correlation between the attentional effects on firing rate and burstiness, this reduction in burstiness was not significantly different from that of the remaining neurons (rank sum test, *P* = 0.8 in overall population, *P* = 0.8 in Monkey N, and *P* = 0.6 in Monkey W). Along similar lines, we also looked at another subset of neurons whose spatial attentional index for firing rate was actually less than that for feature-based attention (black dots in Fig. 4). Again, even in this subset of neurons where the measured spatial attentional effect on firing rate was less than that of feature-based attention, spatial attention was still associated with a significant reduction in burstiness (two monkeys pooled: median = −0.133, *P* = 0.01, *n *= 32, filled black triangle on vertical axis in Fig. 4*A*; Monkey N: median = −0.080, *P* = 0.1, *n *= 16, Monkey W: median = −0.157, *P* = 0.03, *n* = 16). This reduction in burstiness by spatial attention in this subset was not significantly different from that in the remaining neurons (for both monkeys pooled: median = −0.098, *P* = 0.002, rank sum test *P* = 0.8; Monkey N: median = −0.091, *P* = 0.01, rank sum test *P* = 0.7; Monkey W: median = −0.105, *P* = 0.03, rank sum test *P* = 0.98). These results suggest that the significant reduction of burstiness associated with spatial attention (compared with feature-based attention) cannot be explained on the basis of a difference in effect-sizes (as measured by the effects of the two attention types on firing rate).

### Effects of Spike Waveform Duration

Anderson et al. (2013) reported that V4 neurons could be categorized into 2 classes based on a bimodal population distribution of waveform durations, and burstiness effects were only found in the broad-spiking population. Our neuronal population did not show a bimodality in waveform durations (Hartigan's dip test: Monkey N, *P* = 0.4 and Monkey W, *P* = 1; Supplementary Figure 2).

## Discussion

Burstiness, defined as the tendency of a neuron to discharge discrete groups of consecutive action potentials, has been extensively identified from both in vitro and in vivo recordings in various neuron types and brain regions (McCormick et al. 1985; Bair et al. 1994; Compte et al. 2003). Our data demonstrate that spatial attention is associated with a reduction of burstiness in MST neurons. We disambiguate this reduction of burstiness from the concurrent increase in firing rate induced by spatial attention. These results suggest that burstiness reduction might be a ubiquitous effect of spatial attention across visual areas.

The functional properties and neural utility of bursts in spike trains have been a topic of much speculation and interest over the years (Cattaneo et al. 1981; Izhikevich et al. 2003; Krahe and Gabbiani 2004; Shih et al. 2011). Since a burst of spikes may induce a stronger change in postsynaptic potential than more temporally dispersed spikes, it has been suggested that bursts are a more reliable unit to transmit information (Izhikevich 2007). In line with this assertion, other studies have also suggested that bursts enhance functional connectivity between areas (Bonifazi et al. 2009; Kwan and Dan 2012; Womelsdorf et al. 2014). The prevailing hypothesis that bursts enhance information encoding transfer predicts that burstiness would increase when attention is directed towards the RF, so that attended neural responses have an advantage in transmission (Anderson et al. 2013). However, the data indicate the opposite. One possible resolution is that the multiple spikes in a burst carry redundant sensory information: it has been shown that the event rate, where each event is either a single burst of spikes or an isolated spike, is on average a more sensitive measure of direction selectivity than the total number of spikes (Bair et al. 1994). Spatial attention would then make more efficient use of each spike by reducing this redundancy.

A previous study from V4, in the ventral cortical visual processing stream, also reported a reduction of burstiness in the broad-spiking neurons of V4 when spatial attention is directed into their RFs; their median effect size is less than the one we report here for MST (Anderson et al. 2013). One other previous study from V4 (McAdams and Maunsell 1999) also looked at the effect of spatial attention on burstiness. McAdams and Maunsell reported that they found an increase in the rate of bursting with spatial attention, but that this could be accounted for by the higher firing rate with spatial attention. As also pointed out by Anderson et al. (2013), this seemingly contradictory finding was based on a measure of burstiness that increased with firing rate, and the across-neuron analysis used in that study was also less sensitive than the within-neuron analysis that we and Anderson et al. (2013) use. It appears probable that this explains the discrepancy between the findings of McAdams and Maunsell and those of our study as well as Anderson et al. (2013).

Based on their results from area V4, Anderson et al. (2013) proposed a model that accounts for the modulation of firing rate and burstiness by spatial attention via a common cellular mechanism that increases both inhibitory and excitatory synaptic conductances. Our data instead suggest that the effects of spatial attention on burstiness and firing rate stem, at least in part, from separate mechanisms, since in MST the spatial attentional modulation of burstiness and firing rate can be dissociated. This dissociation also argues against another potential explanation for the reduction of burstiness by spatial attention: in a spike train with a refractory period (of say 4 ms), any increase in firing rate will leave the autocorrelation function between time lags of 1 and 4 ms unaffected but will increase the magnitude of the cross-correlation function (shuffle predictor) and therefore, our burstiness measure will decrease with firing rate under these conditions. However, this does not appear to be the case with our results.

In our experiment, monkeys had to maintain fixation within 1.8 degrees of the central fixation point. It is possible that the monkeys made small eye movements within this window during fixation, and that the neural effects of these eye movements (Bair and O'Keefe 1998; Martinez-Conde et al. 2002; Hafed and Krauzlis 2010) interact with the effects of attention to differentially modulate burstiness in the attentional conditions we test. However, due to the low sampling rate of the eye tracker we used (60 Hz), we are unable to perform a reliable analysis of microsaccade effects. Evaluating the role of microsaccades in the burstiness reduction with spatial attention remains a topic for future studies.

Our findings have implications in the context of the feature similarity gain model of visual attention (Treue and Maunsell 1999; Martinez-Trujillo and Treue 2004; Maunsell and Treue 2006), which proposes that the gain of a visual neuron is maximal when the attended feature (or spatial location) matches the neuron's preferred feature (or spatial location). The model supports (but does not require) a unified mechanism for spatial and feature-based attention. In other words, spatial location is just another feature. Our observation of different effects of spatial and feature-based attention on burstiness suggests at least 2 possibilities. Spatial and feature-based attention mechanisms may differ but happen to have qualitatively similar effects on firing rate. Alternatively, both types of attention may share a neural mechanism that modulates firing rate but spatial attention additionally engages a separate mechanism that affects burstiness. Our evidence for, at least partially, separate mechanisms of spatial and feature-based attention is supported by psychophysical studies that have observed differences in the effects of spatial and feature-based attention on human visual perception (Ling et al. 2009) and by recordings from V4 neurons that have observed differences in firing rate modulation by spatial and feature-based attention (Hayden and Gallant 2005; David et al. 2008; Cohen and Maunsell 2011). Furthermore, while the impact of the burstiness reduction by spatial attention on the functional and behavioral consequences of spatial attention remains unknown, our data suggest that the consequences of feature-based attention do not depend on burstiness modulation.

Overall, our results from the dorsal visual pathway suggest that the modulation of burstiness by spatial attention is a general phenomenon across visual cortex that arises from a common neural mechanism. Spatial and feature-based attention, however, may involve partially different underlying neural mechanisms. Further studies of these differences may yield a fuller understanding of the common and unique aspects of various types of attention across visual cortex and how they reflect the underlying attentional neural mechanisms.

## Author Contribution

S.B.R, D.K., and S.T. designed the experiment; S.B.R. and D.K. performed the experiment; C.X. and B.S.K. designed the analysis; C.X. and B.S.K. analyzed the data; C.X., B.S.K. and S.T. wrote the paper

## Supplementary Material

Supplementary material can be found here.

## Supplementary Material

Supplementary Data

Supplementary Data

Supplementary Data
