# Supplementary figures and images for: Spatial Attention Reduces Burstiness in Macaque Visual Cortical Area MST
Source: Cereb Cortex. 2016 Nov 22;27(1):83–91. doi: 10.1093/cercor/bhw326 (PMC5939203; doi:10.1093/cercor/bhw326)

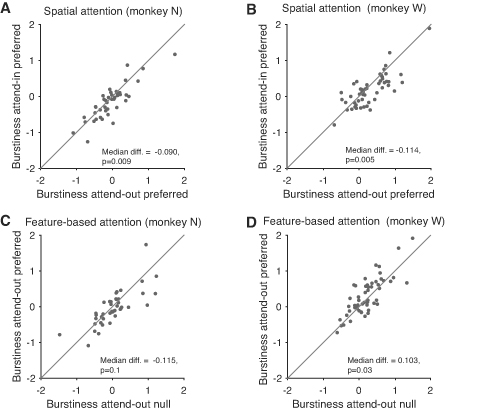

Supplement: Supplementary Data [file figuresupp1.jpeg]

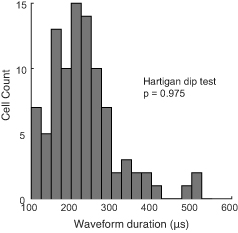

Supplement: Supplementary Data [file figuresupp2.jpeg]
