# Supplementary material for: Spatial Attention Reduces Burstiness in Macaque Visual Cortical Area MST
Source: Cereb Cortex. 2016 Nov 22;27(1):83–91. doi: 10.1093/cercor/bhw326 (PMC5939203; doi:10.1093/cercor/bhw326)
Supplement: Supplementary Data [file suppfiglegends.docx]

Fig. S1. spatial attention significantly reduces burstiness in both monkeys. Scatterplots showing the burstiness effects for the two monkeys: spatial attention reduces burstiness in both monkeys (A,B) while feature-based attention significantly increases burstiness in one monkey (C,D). Each data point represents one neuron and the p-value is the result of a signed-rank test. One data point of (1.7,4.0) was not shown in panel B for visibility. The median difference (median diff.) between the ordinate and abscissa (y-x), and the p-value from a signed rank test are indicated at the bottom-right of each panel.

Fig. S2. No evidence for a bimodal distribution of waveform durations in our dataset. Histogram showing the distribution of waveform durations for all neurons. The p-value from a Hartigan dip test on the distribution is reported in the panel (same test for monkey W, p = 0.4 and for monkey N, p ~ 1 ).
